# Clinical importance of the persistent primitive hypoglossal artery in vascular lesions and endovascular treatment: a narrative review

**DOI:** 10.3389/fsurg.2026.1737542

**Published:** 2026-05-07

**Authors:** Wei Li, Jinlu Yu

**Affiliations:** 1 Department of Neurosurgery, First Hospital of Hebei Medical University, Shijiazhuang, China; 2 Department of Neurosurgery, First Hospital of Jilin University, Changchun, China

**Keywords:** endovascular treatment, persistent primitive hypoglossal artery, vascular lesion, prognosis, review

## Abstract

The persistent primitive hypoglossal artery (PPHA) is a rare and anatomically complex cerebrovascular variant. Although typically asymptomatic, its presence is associated with various cerebrovascular pathologies, including intracranial aneurysms, carotid artery stenosis, acute large vessel occlusion, moyamoya disease, brain arteriovenous malformations, and other vascular anomalies. When such lesions involve the PPHA, therapeutic intervention may be necessitated. Surgical management is particularly challenging due to the artery's deep anatomical location and intricate surrounding vasculature. Consequently, endovascular therapy (EVT) has emerged as a preferable alternative to open surgery, offering a favorable safety profile and reduced technical complexity. Despite this, a substantial need remains in the literature regarding systematic evaluations of the PPHA's clinical significance in vascular pathology and the efficacy of EVT. This review aims to address this need through a comprehensive narrative synthesis of available literature and clinical experience in managing these complex cases. This review found that when EVT is required, the PPHA can serve as an access route. However, given that it often provides the sole blood supply to the posterior circulation—particularly in the context of bilateral hypoplastic vertebral arteries—the vessel must be meticulously preserved during interventions for associated conditions.

## 1 Introduction

The persistent primitive hypoglossal artery (PPHA) is the second most prevalent persistent primitive artery, with a prevalence of 0.03%–0.3%, following the persistent primitive trigeminal artery (0.5%–0.7%) (1). Historically, the PPHA was termed either the last occipital intersegmental artery or last hypoglossal intersegmental artery (2, 3). The PPHA results from the failure of the embryonic hypoglossal artery to regress during development. The PPHA may originate from the internal carotid artery (ICA), external carotid artery (ECA), or common carotid artery (CCA). It traverses the hypoglossal canal to connect with the vertebrobasilar system, or it may terminate as a variant of the posterior inferior cerebellar artery (PICA) (4–6). The PPHA supplements for the vertebrobasilar system.

The PPHA is typically an incidental finding discovered during imaging for unrelated indications (7). It may also represent a vascular pathology arising secondarily due to hemodynamic alterations. Additionally, the presence of this persistent artery can have significant clinical implications, including intracranial aneurysm (8), carotid artery stenosis (9), acute large vessel occlusion (10), moyamoya disease (MMD) (11), brain arteriovenous malformation (AVM) (12), hypoglossal nerve palsy (13), a pulsatile neck mass (14), carotid cavernous fistula (15), and other vascular anomalies (16).

Certain vascular lesions involving the PPHA may necessitate intervention due to their pathological nature. The surgical management of such lesions is significantly complicated by the PPHA's deep anatomical location and intricate vascular architecture. In contrast, endovascular treatment (EVT) has emerged as a safer and technically less demanding alternative to open surgical approaches. However, there remains a need in the literature regarding comprehensive reviews assessing the clinical significance of PPHA involvement in vascular lesions and the efficacy of EVT. This review addresses this need by integrating the literature with clinical experience in managing these complex cases.

## 2 Methods and results

### 2.1 Search strategy and data sources

A systematic search of the PubMed database was performed to identify English-language articles relevant to the PPHA. The primary search term employed was “PPHA”, with no additional keywords or filters. Article types considered for inclusion comprised case reports, case series, prospective studies, review articles, and other relevant studies. To ensure comprehensiveness and minimize the risk of missing relevant studies, the reference lists of all retrieved articles were meticulously hand-searched for potentially eligible studies not captured by the initial database query.

### 2.2 Selection criteria

**Figure 1:**
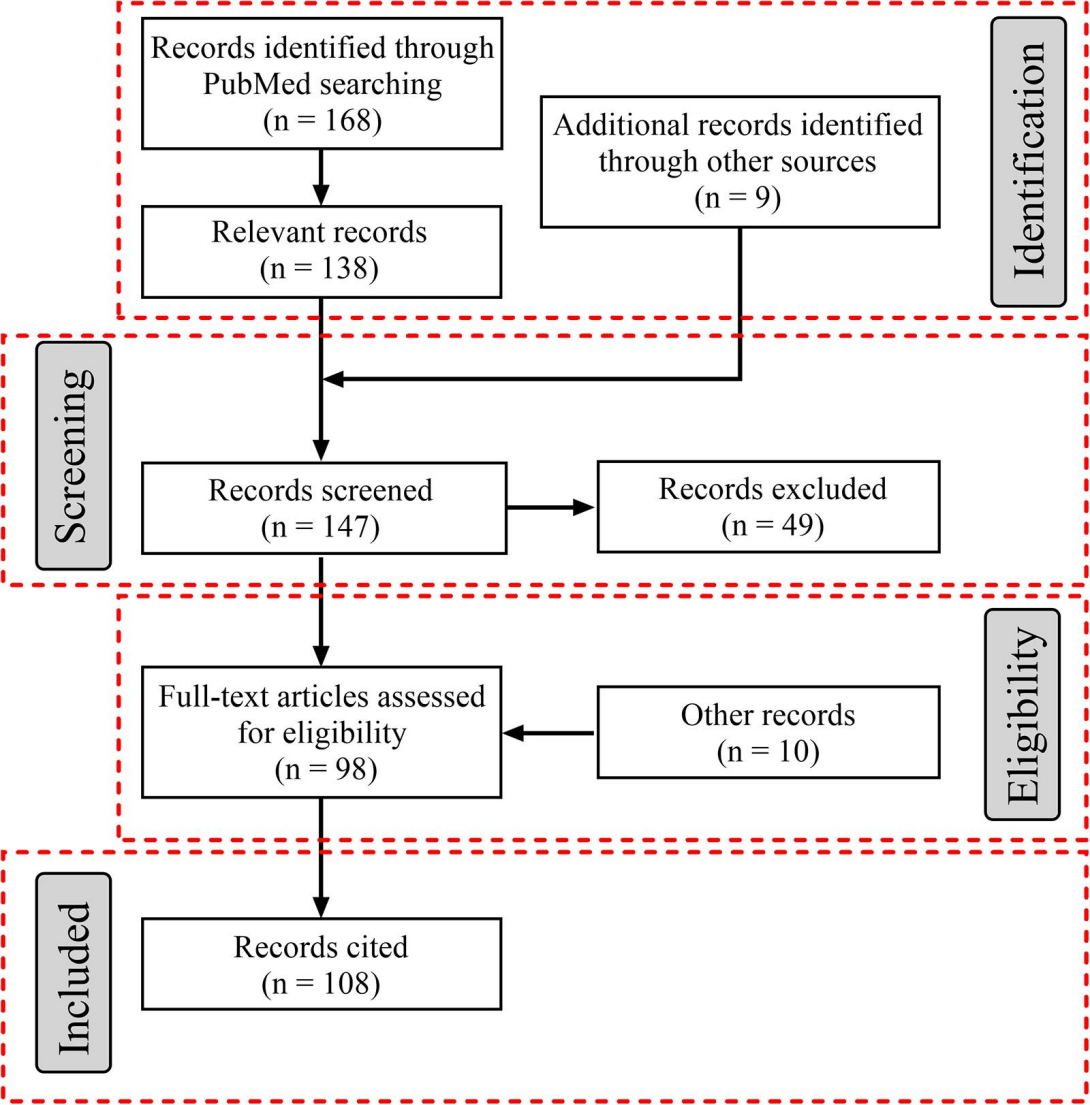
Flow chart of the literature search.

Studies were considered eligible for inclusion if they met the following criteria: (a) availability of the full text or sufficient extractable data; and (b) content pertaining to the anatomy and variants of the PPHA, or its involvement in vascular pathologies and endovascular interventions. Publications lacking adequate information were excluded. Following the screening process, a total of 108 relevant articles were cited in the present review. The study selection process is summarized in a flowchart (Figure 1).

### 2.3 Discussion contents

In 2025, Elsayed et al. proposed a classification system defining six categories of PPHA: (1) isolated asymptomatic PPHA; (2) PPHA with stenosis; (3) aneurysmal PPHA; (4) PPHA with carotid artery stenosis; (5) PPHA with cranial nerve compression; and (6) PPHA associated with other vascular anomalies (16). The present review builds on and expands this framework by discussing the following aspects of PPHA: angiographic anatomy and variants; its role in intracranial aneurysm, carotid artery stenosis, acute large vessel occlusion, MMD, and brain AVM; and other clinical implications.

## 3 Angiographic anatomy and variant

**Figure 2:**
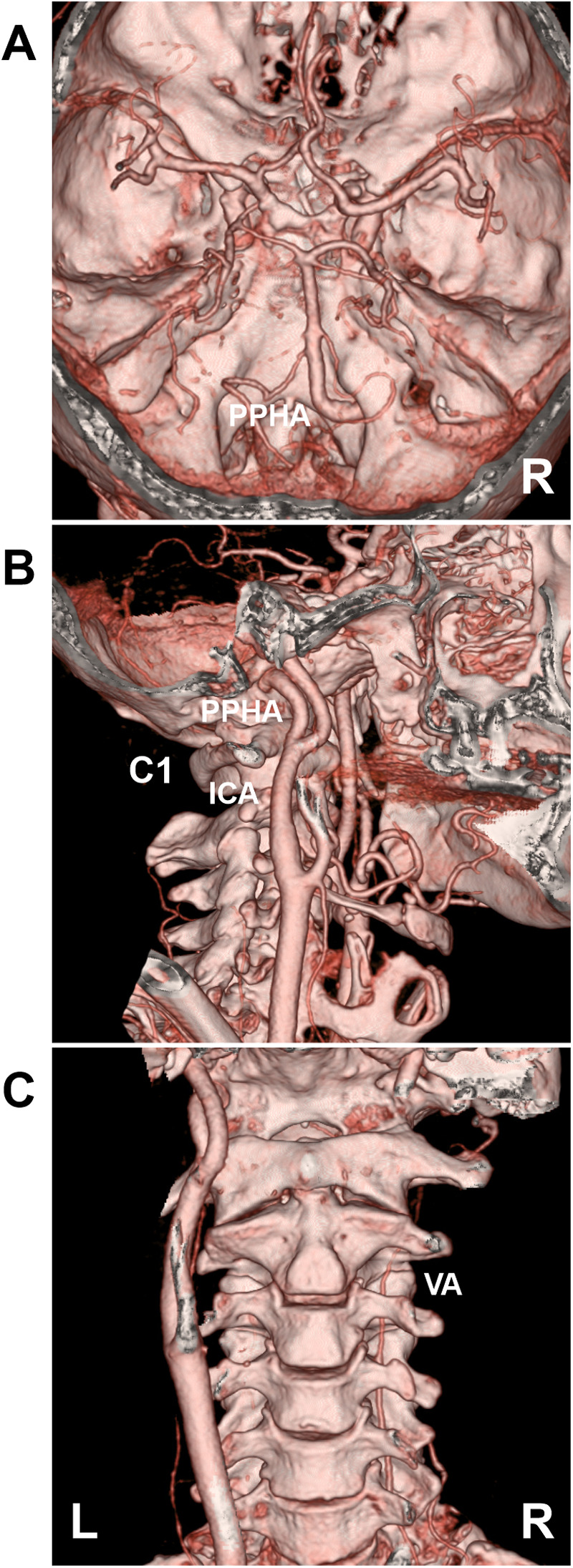
Angiographic anatomy of the PPHA on CTA. **(A)** CTA shows a right PPHA passing through the hypoglossal canal to replace the ipsilateral VA and joins the basilar artery. **(B)** CTA shows the PPHA arising from the C1 level of the ICA and ascending through the hypoglossal canal. **(C)** CTA shows contralateral (left) VA absence with ipsilateral (right) VA hypoplasia. C1, first cervical vertebra; CTA, computed tomography angiography; ICA, internal carotid artery; L, left; PPHA, persistent primitive hypoglossal artery; R, right; VA, vertebral artery.

Because the definitive diagnostic criterion for PPHA is its passage through the hypoglossal canal (3, 6), computed tomography angiography is essential for confirming its anatomical course (Figure 2).

### 3.1 PPHA anatomy

**Figure 3:**
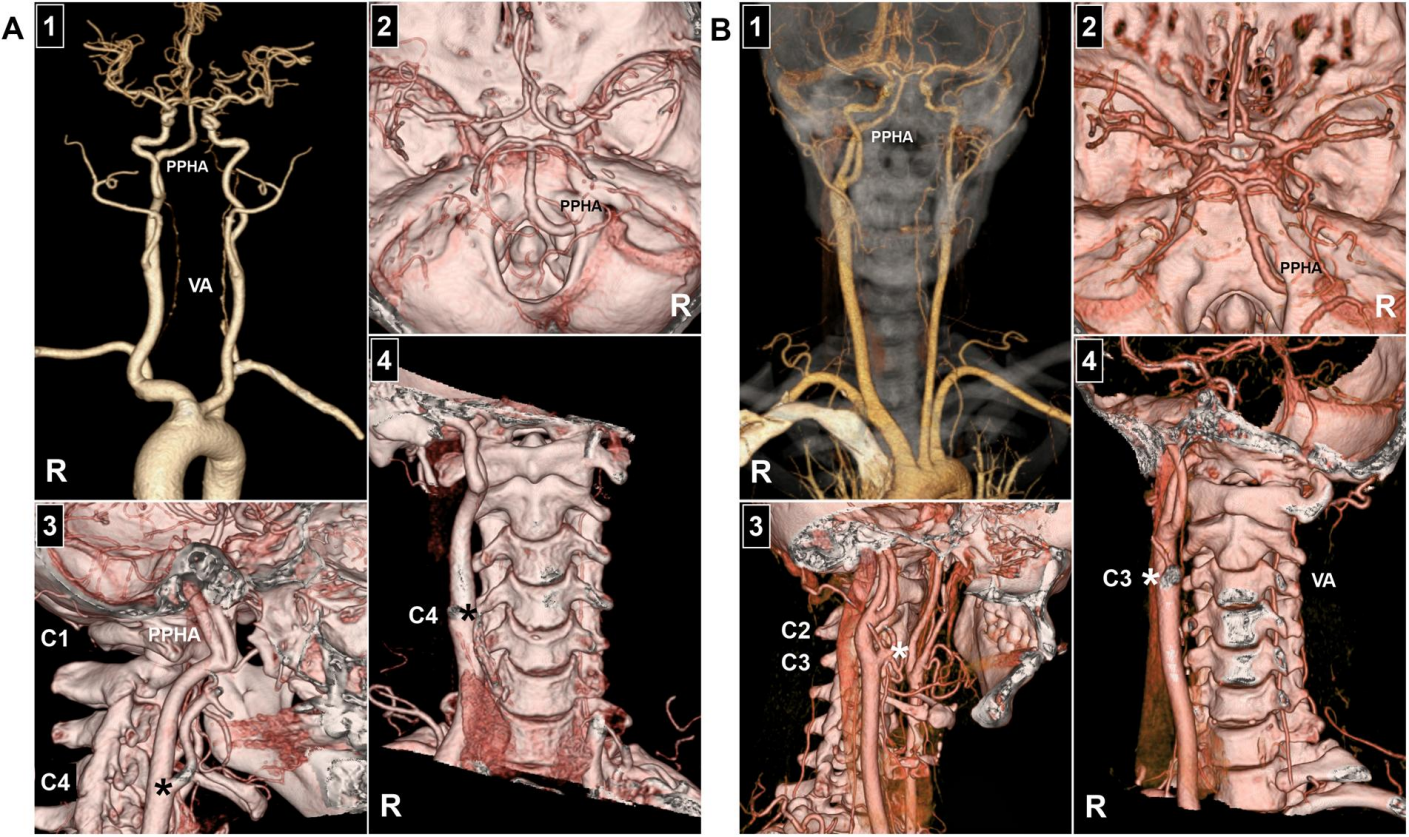
Development of VA and PcomA in PPHA. **(A)** Panel 1: Head and neck CTA reveals a right PPHA with bilateral VA hypoplasia and absence of PcomAs. Panel 2: Head CTA demonstrates a right PPHA coursing through the hypoglossal canal to replace the ipsilateral VA and join the basilar artery. Panels 3–4: Neck CTA images show the PPHA originating from the C1 level of the ICA, with the carotid bifurcation located at the C4 level (asterisks). **(B)** Panel 1: Head and neck CTA reveals a right PPHA with bilateral VA hypoplasia and absence of PcomAs. Panel 2: Head CTA demonstrates a right PPHA passing through the hypoglossal canal to replace the ipsilateral VA and join the basilar artery. Panels 3–4: Neck CTA images show the PPHA arising from the C2 level of the ICA, with the carotid bifurcation at the C3 level (asterisks). C1, C2, C3, C4: first, second, third, and fourth cervical vertebrae, respectively; CTA, computed tomography angiography; ICA, internal carotid artery; PcomA, posterior communicating artery; PPHA, persistent primitive hypoglossal artery; R, right; VA, vertebral artery.

**Figure 4:**
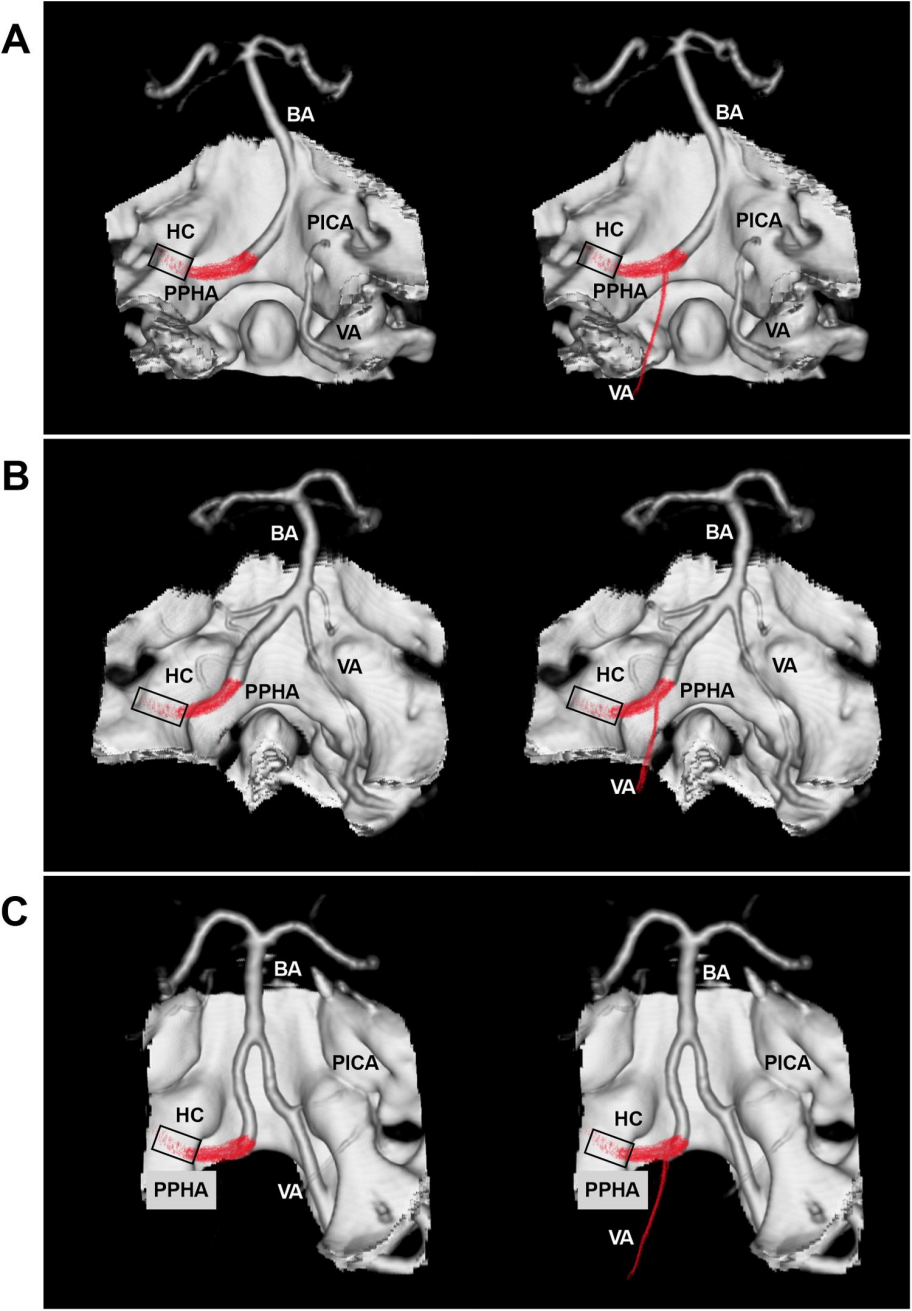
Relationship between the PPHA and VA on hybrid CTA. In all panels, hybrid CTA images show the PPHA passing through the HC to replace the VA. **(A)** The contralateral VA is hypoplastic and terminates in the PICA; the ipsilateral VA is absent (left panel) or hypoplastic (right panel). **(B)** The contralateral hypoplastic VA connects to the BA; the ipsilateral VA is absent (left panel) or hypoplastic (right panel). **(C)** The contralateral VA is normal; the ipsilateral VA is absent (left panel) or hypoplastic (right panel). BA, basilar artery; CTA, computed tomography angiography; HC, hypoglossal canal; PICA, posterior inferior cerebellar artery; PPHA, persistent primitive hypoglossal artery; VA, vertebral artery.

Similar to the classification of proatlantal intersegmental arteries, Uchino et al. proposed categorizing PPHAs into two types: type 1, arising from the ICA, and type 2, originating from the ECA (3). Rarely, bilateral PPHAs may occur, presenting as either side-dominant or symmetric (17–21). The PPHA typically correlates with a hypoplastic or absent vertebral artery (VA) or posterior communicating artery (PcomA) (Figure 3). Agnoli et al. reviewed 27 PPHAs and found that only 4% had bilaterally normal VAs, compared with 79% showing bilateral or ipsilateral VA hypoplasia. Similarly, bilaterally normal PcomAs were seen in only 4%, whereas 58% had bilateral PcomA hypoplasia (22). The anatomical relationship between the PPHA and bilateral VAs was illustrated in Figure 4.

#### 3.1.1 PPHA arising from ICA

The PPHA originating from the ICA at C1–C3 vertebral levels constitutes the classic anatomical variant, with Varvari et al.'s review of 57 cases confirming this ICA origin pattern in 89.5% (51/57) of instances (23). Rarely, the PPHA can originate from the cervical ICA close to the carotid bifurcation (24). It runs parallel to the posterior surface of the ICA, joins the 12th cranial nerve, then passes through the enlarged hypoglossal canal with variable tortuosity to enter the posterior cranial fossa (25). Its termination may occur at either the VA or as a complete VA replacement joining the basilar artery (BA) (5, 6, 26, 27).

#### 3.1.2 PPHA arising from ECA or CCA

Varvari et al.'s review demonstrated an ECA origin in 8.8% of cases and a CCA origin in 1.8% of cases (23). The PPHA with ECA origin demonstrates an extracranial course resembling that of the ascending pharyngeal artery (AphA) (28, 29). The PPHA can have a common trunk with the occipital artery (30). In such cases, the PPHA may represent a significantly dilated hypoglossal branch of the AphA before traversing the hypoglossal canal to enter the posterior cranial fossa to join the VA (3). When the PPHA arises from the CCA, it courses along the posterior surface of the ICA, joins the hypoglossal nerve, and passes through the hypoglossal canal into the posterior cranial fossa (31). Rarely, when the PPHA arises from the CCA, the bilateral ICAs may be absent, with the contralateral VA being hypoplastic, allowing the PPHA to serve as the sole blood supply to the brain (32).

### 3.2 PPHA variant

A rare variant of the PPHA is the PPHA-PICA supply, which occurs when the PICA arises from the ICA or ECA (33, 34). Morris and Moffat described the embryonic hypoglossal artery as having three components: (a) the hypoglossal artery proper; (b) a segment of the primitive lateral basilar–vertebral anastomosis (PLBA); and (c) transverse anastomotic channels linking the PLBA to the longitudinal neural artery. Caudally, the PLBA connects with the dorsal branch of the pro-atlantal intersegmental artery; rostrally, it gives off several branches. One of these branches eventually forms the PICA (35).

When the PICA arises from the ICA, the vessel runs parallel to the posterior surface of the ICA (36–38). When the PICA arises from the ECA, the vessel may represent a significantly dilated hypoglossal branch of the AphA (3, 39). After passing through the hypoglossal canal, the vessel enters the posterior fossa to continue directly as the PICA, without an intervening segment of the VA or BA.

## 4 Role of the PPHA in intracranial aneurysm

**Figure 5:**
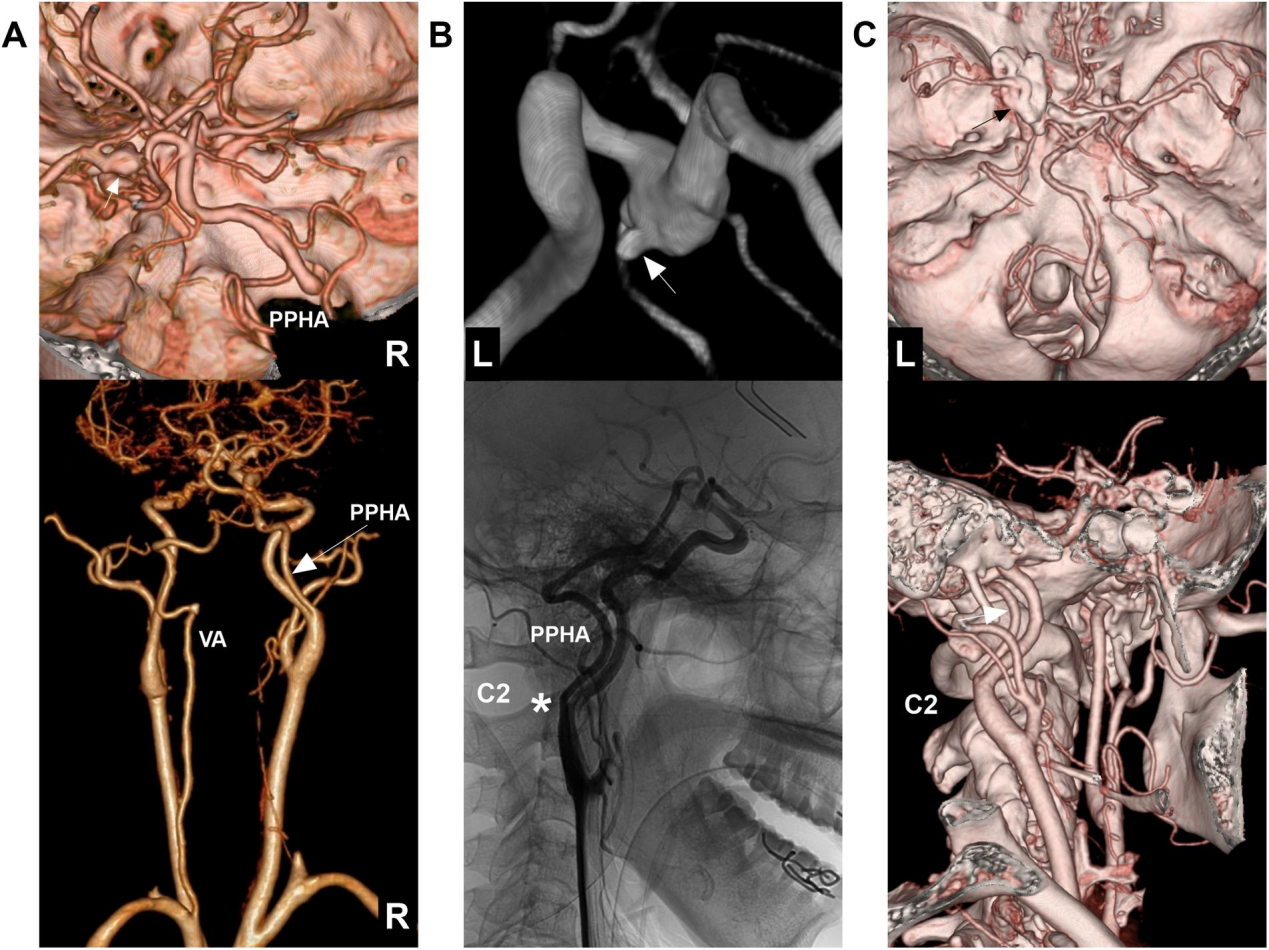
PPHA with anterior circulation aneurysm. **(A)** Upper panel: CTA demonstrates a right PPHA passing through the hypoglossal canal and a left PcomA aneurysm (arrow). Lower panel: CTA reveals the right PPHA with ipsilateral hypoplastic VA and a normal contralateral extracranial VA. **(B)** Upper panel: DSA displays the PcomA aneurysm with a bleb (arrow). Lower panel: DSA illustrates the PPHA arising from the ICA at the C2 level (asterisk). **(C)** Upper panel: CTA confirms clipping of the left PcomA aneurysm (arrow). Lower panel: CTA shows the PPHA (arrow). C2, second cervical vertebra; CTA, computed tomography angiography; DSA, digital subtraction angiography; L, left; PcomA, posterior communicating artery; PPHA, persistent primitive hypoglossal artery; R, right; VA, vertebral artery.

In cases with PPHA, the PPHA often serves as the sole blood supply to the posterior circulation. This leads to increased hemodynamic instability and elevated wall shear stress gradients in both the PPHA and posterior circulation (40, 41). Additionally, the PPHA may be associated with anomalous vessel wall structure (10, 40, 42, 43). These factors can predispose to aneurysm formation on the PPHA itself and within the posterior circulation. Additionally, when a PPHA exists, there may be abnormal hemodynamics in the anterior circulation, leading to aneurysm formation in this territory (Figure 5) (22, 44, 45).

Previous studies reported that 22%–26% of PPHAs had associated aneurysms, with 85% located on the PPHA itself or in the posterior circulation (26, 40, 44, 46–48). The PPHA-BA junction was most common, followed by the PPHA-PICA junction (26, 49–51). In Yamamoto et al.'s review of 11 PPHA-associated aneurysms, 81.8% were located at the PPHA-BA junction and 18.2% at the PPHA-PICA junction (52). Other sites included the BA-anterior inferior cerebellar artery junction, BA-superior cerebellar artery junction, BA tip, posterior cerebral artery (PCA), even in the fenestration (23, 43, 49, 52–56). PPHA-related aneurysms may manifest as saccular, dissecting, or fusiform types, and can be single or multiple (18, 26, 44, 57, 58). The anatomical location of these aneurysms and their proximity to the surrounding venous system significantly complicate surgical clipping (49, 52, 54).

**Figure 6:**
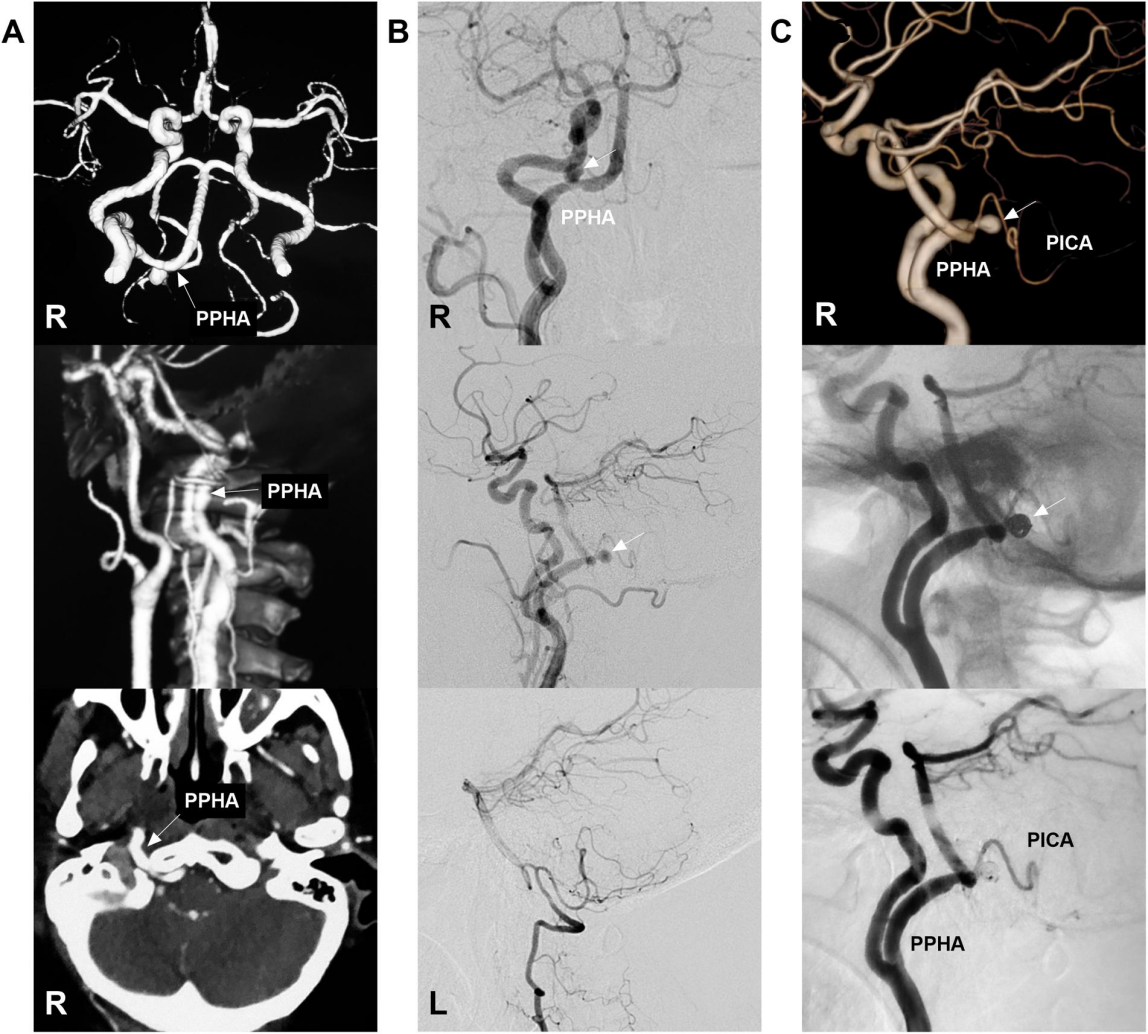
Coiling an aneurysm at the junction of PPHA and PICA. **(A)** Upper and middle panels: CTA (upper) and CTA with semi-transparent bone reconstruction (middle) demonstrate a right PPHA (arrows) extending into the posterior cranial fossa. Lower panel: Maximum intensity projection of CTA reveals the PPHA (arrow) coursing through the hypoglossal canal. **(B)** Upper and middle panels: DSA images of the right carotid artery in anteroposterior (upper) and lateral (middle) views show an aneurysm at the junction of the PPHA and PICA (arrows). Lower panel: DSA of the left VA demonstrates a hypoplastic VA. **(C)** Upper panel: Three-dimensional DSA displays the saccular aneurysm (arrow). Middle panel: Unsubtracted DSA confirms the aneurysm (arrow) was coiled via the PPHA. Lower panel: Post-procedural DSA confirms patency of the PPHA and PICA. CTA, computed tomography angiography; DSA, digital subtraction angiography; L, left; PICA, posterior inferior cerebellar artery; PPHA, persistent primitive hypoglossal artery; R, right; VA, vertebral artery.

EVT has demonstrated favorable safety and efficacy as a therapeutic approach for these aneurysms (40, 46, 59, 60). For anterior circulation aneurysms, EVT followed the same standard principles comparable to cases without PPHA involvement. Saccular aneurysms associated with PPHA were amenable to coil embolization, while wide-necked, dissecting, or fusiform aneurysms benefited from stent-assisted coiling, balloon-assisted coiling, or flow diverter deployment (Figure 6) (51, 59–62). However, navigating modern flow diverters such as the Pipeline embolization device (Medtronic, Irvine, CA, USA) and Surpass Evolve flow diverter (Stryker Neurovascular, Fremont, CA) through the tortuous hypoglossal canal presents specific challenges. Due to the rarity of PPHA, no reports of flow diverter deployment via this route currently exist; nevertheless, this possibility warrants consideration.

As PPHA can be the sole arterial supply to posterior circulation, iatrogenic injury carries risk of posterior circulation ischemia, leading to significant morbidity and mortality. Therefore, the PPHA must be preserved.

## 5 Role of the PPHA in carotid artery stenosis

The PPHA may be associated with carotid artery stenosis, most commonly involving the ICA beneath the PPHA (7, 63, 64). Additionally, stenosis may occur at the CCA near the carotid bifurcation, the PPHA itself, or both the PPHA and ICA (9, 23, 65, 66). In PPHA patients, ICA and CCA stenosis may reduce cerebral perfusion or cause embolic infarctions in both circulation territories (63–65, 67, 68). Notably, when PPHA coexists with bilateral hypoplastic VAs and PcomAs, the posterior circulation becomes dependent on the ICA. This hemodynamic vulnerability necessitates intervention.

For PPHAs with carotid artery stenosis, carotid endarterectomy (CEA) and carotid artery stenting (CAS) are available (69, 70). CAS may be preferable for preventing further ischemic attacks in patients with PPHA, as ICA stenosis typically occurs between the carotid bifurcation and the PPHA origin—a region where shunt placement during CEA is technically challenging due to the complex anatomy and high cervical location (63, 64). In Burgard et al.'s review, CAS was successfully performed in 11 PPHA patients (64). However, CAS in this context poses significant challenges due to the risk of arterial embolization, which may lead to ischemic strokes in both anterior and posterior circulations.

The CAS for carotid artery stenosis involving the PPHA requires careful evaluation of several factors: the location of stenosis relative to the PPHA, the choice of embolic protection strategy, and the preservation of blood flow to both anterior and posterior circulations (71). For ICA stenosis above the PPHA origin, a single distal protection device placed in the distal ICA may suffice. When stenosis involves the PPHA distal to its origin, a single distal protection device may also be sufficient during stenting (66). Concurrent ICA and PPHA stenosis often necessitates double-distal protection during balloon angioplasty or stenting (72, 73). In cases of carotid artery stenosis below the PPHA, CAS may involve a distal protection device below the PPHA if the distance between the stenosis and the PPHA origin is sufficient, or dual embolic protection devices may be necessary for both the ICA and PPHA (74, 75).

In addition to distal umbrella-like protective devices, a balloon protection system can be employed. The Mo.Ma proximal flow blockade and distal protection system (Medtronic Invatec, Braunfeld, Switzerland) can stop blood flow in the CCA, ICA, or ECA using two balloons, thereby selectively preserving the PPHA. Blood is withdrawn through the Mo.Ma device after each balloon predilation for stenosis, preventing emboli from entering the anterior and posterior circulations (76–78). Similarly, the Gore flow-reversal system (WL Gore and Associates, Flagstaff, AZ, USA) can also be used, as it protects against embolization by occluding both proximal and distal carotid arteries (66, 79).

However, in patients with a PPHA, balloon-induced flow occlusion carries potential ischemic risks to both the anterior and posterior circulations. If the PPHA is the sole arterial supply to the posterior circulation, inflating a balloon in the CCA, ICA, or ECA could precipitate immediate brainstem ischemia due to the physiological consequences of proximal occlusion. This risk is further compounded by the frequent absence of sufficient collateral flow. Therefore, thorough collateral flow evaluation—including preoperative angiography, balloon occlusion testing, and cross-flow studies—is essential before this procedure to identify patients at increased risk and guide protective strategies (65).

## 6 Role of the PPHA in acute large vessel occlusion

In cases with a PPHA, thrombus behavior directly determines stroke distribution: if the thrombus spares the PPHA and embolizes into the anterior circulation, it will cause an anterior circulation stroke; if the thrombus travels through the PPHA into the posterior circulation, it will lead to a posterior circulation stroke. During a thrombus event, it's necessary to recognize that a PPHA could be one of the causes of a simultaneous acute infarction in both the anterior and posterior vascular territories (10, 80–84).

Thrombi may be cardioembolic, atherosclerotic or dissecting, with cardioembolism being the likely cause (85, 86). High thrombus burden can precipitate acute large vessel occlusion, necessitating urgent intervention. The trials demonstrate mechanical thrombectomy (MT) achieves high recanalization rates with acceptable complication profiles (87–89). When thrombus is limited to the anterior circulation without extension into the PPHA, MT can follow standard procedures comparable to cases without PPHA involvement (90). Successful MT requires access to the thromboembolic region while preventing thrombus migration into the PPHA during the procedure (82). When the posterior circulation is involved in an acute large vessel occlusion, the PPHA must be utilized as the EVT access route (10). It is equally critical to avoid thrombus entry into the ICA. Several cases of MT via the PPHA have been reported, including those by Park et al. (acute BA occlusion) (10) and See et al. (acute BA occlusion) (85); all achieved successful recanalization. If both the anterior and posterior circulations—supplied via the PPHA and ICA—are affected by emboli, simultaneous MT may be necessary. In addition, aspiration thrombectomy via the PPHA can also be utilized (80).

Rarely, the PPHA itself and/or its parent artery (ICA, CCA, or ECA) may be occluded (91). When this occurs, MT becomes complex. A balloon or distal protective device in an unoccluded large vessel can prevent thrombus migration into the anterior or posterior circulation during aspiration or MT. If both the PPHA and its parent artery are occluded, after reopening one vessel, the reopened vessel should be protected. In addition to a distal protective device, a balloon guide catheter can be utilized (83).

## 7 Role of the PPHA in MMD

The coexistence of PPHA and MMD is uncommon, and the exact pathogenesis of this combination remains unclear (11, 92). Two potential explanations have been proposed: (1) there may exist a congenital developmental link between these conditions, or (2) this may represent a chance occurrence without direct causality. Notably, the period when PPHA disappears at the embryonic stage (5–14 mm) almost corresponds to the period (11–14 mm) when the vascular state is similar to moyamoya phenomena (11). MMD may exert hemodynamic stress on the PPHA, disturbing its spontaneous closure (11, 93). Therefore, there may be a close relationship between MMD and PPHA, not just a coincidence.

When both conditions coexist, the PPHA can become the primary blood supply to the brain (94). As the main source of cerebral blood flow, the PPHA may lead to the formation and rupture of a BA bifurcation aneurysm under high hemodynamic stress (95). In such cases, EVT may be necessary. For MMD patients with PPHA, anterior circulation insufficiency might require revascularization. In Kurose et al.'s report, a 44-year-old female with symptomatic MMD and PPHA arising from the ICA underwent bilateral superficial temporal artery-middle cerebral artery anastomosis, resulting in a good prognosis (96).

## 8 Role of the PPHA in brain AVM

Rarely, brain AVMs may coexist with PPHA. Recently, brain AVMs are no longer considered congenital vascular anomalies; therefore, their coexistence with PPHA is likely incidental (97). The reported coexistence rate is approximately 3% in patients with PPHA (98). Brain AVMs associated with PPHA may be located in infratentorial or supratentorial regions (98–102). When an AVM is located in the supratentorial region with only anterior circulation supply, the PPHA was not associated with the AVM (101, 103). When brain AVMs are located in the occipital lobe, cerebellum, or brainstem, they can be fed by the PCA or vertebrobasilar arterial system, which are derived from the PPHA (102).

In these brain AVMs, the PPHA can serve as an EVT pathway for the AVM or its associated flow-related aneurysm. In Shibata et al.'s report, after 90% of the cerebral AVM was embolized via the PPHA and PCA, complete resection was subsequently performed (104). Similarly, Gupta et al. reported a thalamo-capsular AVM supplied by the PPHA; after most of the AVM was embolized via the PPHA and PCA perforating feeders, stereotactic radiotherapy was administered (100). Pavlisa et al. reported a 61-year-old female with a cerebellar AVM in whom a ruptured dissecting aneurysm on the feeding artery of the anterior inferior cerebellar artery (AICA) was embolized via the PPHA and AICA (102).

When treating brain AVMs via the PPHA, special consideration is required because the PPHA often serves as the sole blood supply to the posterior circulation, which typically lacks PcomAs and VAs. Therefore, preservation of the PPHA is essential to maintain adequate perfusion of the posterior circulation.

## 9 Other roles

### 9.1 Hypoglossal nerve compression by PPHA

As the main artery supplying the posterior circulation, the PPHA may be subject to increased hemodynamic pressure. When the PPHA undergoes dilation or aneurysmal change, or in patients with hypertension, it may compress the hypoglossal nerve, leading to neuropathy (13, 105, 106). Hikichi et al. reported a case of hypoglossal nerve palsy in a 51-year-old patient who had a PPHA exhibiting irregular caliber as it passed through the hypoglossal canal (13). Meila et al. described a similar case in a 71-year-old patient, in whom a calcified PPHA was identified as the putative cause of neurovascular compression syndrome (106). Treatment options for PPHA-induced hypoglossal nerve compression are limited. While surgical decompression of the hypoglossal canal is theoretically effective, the canal's deep location and complex anatomy make the procedure challenging.

Theoretically, flow diverter deployment may be considered for an aneurysmal PPHA, although no such cases have been reported to date. Therefore, conservative treatment is often required. In hypertensive patients, antihypertensive therapy may mitigate the neurovascular compression syndrome, leading to symptom improvement (105). In the case reported by Hikichi et al., the patient's symptoms resolved within six months of onset, and tongue weakness nearly fully resolved by one year (13). In addition, carbamazepine may be effective. In the case reported by Meila et al., the patient remained asymptomatic at three-month follow-up after treatment with carbamazepine (106).

### 9.2 Steal phenomena

PPHA may exhibit reversal of blood flow from the posterior into the anterior circulation, which could be a cause of posterior circulation insufficiency (107). In Garge et al.'s report, a case with bilateral PPHAs was described in which the right ICA was occluded, and the right VA supplied the anterior circulation via the right PPHA (108). In Elhammady et al.'s report, the distal right VA connected with a PPHA to supply the severely stenotic right ICA in a retrograde fashion, reconstituting antegrade flow in the distal cervical ICA (107). In such cases, EVT may be necessary to recanalize the ICA.

## 10 Summary

PPHA is a rare cerebrovascular variant with complex anatomy. It is typically asymptomatic. However, it may be associated with various cerebrovascular pathologies, including intracranial aneurysm, carotid artery stenosis, acute large vessel occlusion, MMD, brain AVM, as well as hypoglossal nerve palsy and other vascular anomalies. When these conditions require treatment, EVT can be a viable option, and the PPHA may serve as a potential access route. However, since the PPHA often provides hemodynamically critical blood supply, it must be preserved whenever possible during any intervention.

## References

[1] Namba K. Persistent primitive arteries. No Shinkei Geka. (2024) 52(3):549–59. 10.11477/mf.1436204948 38783498

[2] Padget DH. Designation of the embryonic intersegmental arteries in reference to the vertebral artery and subclavian stem. Anat Rec. (1954) 119(3):349–56. 10.1002/ar.1091190306 13197795

[3] Uchino A Saito N Okada Y Kozawa E Nishi N Mizukoshi W Persistent hypoglossal artery and its variants diagnosed by CT and MR angiography. Neuroradiology. (2013) 55(1):17–23. 10.1007/s00234-012-1074-0 22821359

[4] Stern J Correll JW Bryan N. Persistent hypoglossal artery and persistent trigeminal artery presenting with posterior fossa transient ischemic attacks. Report of two cases. J Neurosurg. (1978) 49(4):614–9. 10.3171/jns.1978.49.4.0614 690694

[5] Vasović L Milenković Z Jovanović I Cukuranović R Jovanović P Stefanović I. Hypoglossal artery: a review of normal and pathological features. Neurosurg Rev. (2008) 31(4):385–95. 10.1007/s10143-008-0145-5 18548302

[6] Brismar J. Persistent hypoglossal artery, diagnostic criteria. Report of a case. Acta Radiol Diagn (Stockh). (1976) 17(2):160–6. 10.1177/028418517601700204 1274653

[7] Madden NJ Calligaro KD Dougherty MJ Maloni K Troutman DA. Persistent hypoglossal artery: challenges associated with carotid revascularization. Vasc Endovascular Surg. (2019) 53(7):589–92. 10.1177/1538574419859102 31248350

[8] Tse GH Martin A Dyde RA Coley SC. Persistent hypoglossal artery aneurysm: case report and qualitative systematic review. Interv Neuroradiol. (2019) 25(2):164–71. 10.1177/1591019918809087 30394836 PMC6448380

[9] Kawamura K Tokugawa J Watanabe M Fujita N Teramoto S Kimura T Persistent primitive hypoglossal artery with ipsilateral symptomatic carotid artery stenosis and cerebral aneurysm. J Stroke Cerebrovasc Dis. (2021) 30(11):106099. 10.1016/j.jstrokecerebrovasdis.2021.106099 34536812

[10] Park JS Shin BS Kang HG. Endovascular treatment for acute basilar artery occlusion via persistent primitive hypoglossal artery: a case report. Medicine (Baltimore). (2021) 100(48):e27998. 10.1097/MD.0000000000027998 35049208 PMC9191366

[11] Komiyama M Nakajima H Nishikawa M Yasui T Kitano S Sakamoto H High incidence of persistent primitive arteries in moyamoya and quasi-moyamoya diseases. Neurol Med Chir (Tokyo). (1999) 39(6):416–20. 10.2176/nmc.39.416 10396115

[12] Kageyama H Toyooka T Osada H Tsuzuki N. Infratentorial arteriovenous malformation associated with persistent primitive hypoglossal artery. Surg Neurol Int. (2015) 6:71. 10.4103/2152-7806.156633 25984385 PMC4427814

[13] Hikichi H Ueno T Iwamura M Nishijima H Arai A Suzuki C Hypoglossal nerve palsy due to compression by a persistent primitive hypoglossal artery: case report. J Stroke Cerebrovasc Dis. (2020) 29(2):104459. 10.1016/j.jstrokecerebrovasdis.2019.104459 31839548

[14] Fox T Tsouknidas I Tong RT Hirsch H. Persistent primitive hypoglossal artery presenting as a pulsatile neck mass. J Vasc Surg. (2025) 82(5):1878–9. 10.1016/j.jvs.2024.12.036 39706229

[15] Gerlach J Jensen HP Spuler H Viehweger G. Traumatic carotico-cavernous fistula combined with persisting primitive hypoglossal artery. J Neurosurg. (1963) 20:885–7. 10.3171/jns.1963.20.10.0885 14186083

[16] Elsayed NA Ali AA. Persistent primitive hypoglossal artery: a classification-based approach to understanding and managing a rare vascular anomaly. Acta Neurochir (Wien). (2025) 167(1):279. 10.1007/s00701-025-06654-w 41128837 PMC12549736

[17] Choudhary G Ahuja K Khan R Kubal W. Bilateral persistent primitive hypoglossal artery presenting with hemiplegia(✰). Radiol Case Rep. (2018) 13(5):1072–5. 10.1016/j.radcr.2018.04.022 30228846 PMC6137901

[18] Murayama Y Fujimoto N Matsumoto K. Bilateral persistent primitive hypoglossal arteries associated with a large ruptured aneurysm on one side. Surg Neurol. (1985) 24(5):498–502. 10.1016/0090-3019(85)90263-0 4049224

[19] Ozawa M Uchino A Saito N Maruyama H. Bilateral persistent hypoglossal arteries: a case report and literature review. Surg Radiol Anat. (2019) 41(9):1083–5. 10.1007/s00276-019-02243-6 31016350

[20] Ren X. Posterior fossa transient ischemic attack in the setting of bilateral persistent hypoglossal arteries: a case report and literature review. Medicine (Baltimore). (2021) 100(45):e27875. 10.1097/MD.0000000000027875 34766601 PMC10545121

[21] Takahashi H Tanaka H Fujita N Tomiyama N. Bilateral persistent hypoglossal arteries: MRI findings. Br J Radiol. (2012) 85(1010):e46–8. 10.1259/bjr/21939976 22308227 PMC3473955

[22] Agnoli AL. Vascular anomalies and subarachnoid haemorrhage associated with persisting embryonic vessels. Acta Neurochir (Wien). (1982) 60(3-4):183–99. 10.1007/BF01406306 7072535

[23] Varvari I Bos EM Dinkelaar W van Es AC Can A Hunfeld M Fatal subarachnoid hemorrhage from an aneurysm of a persistent primitive hypoglossal artery: case series and literature overview. World Neurosurg. (2018) 117:285–91. 10.1016/j.wneu.2018.06.119 29940384

[24] Suzuki S Uchino A Numaguchi Y. Low origin of the persistent hypoglossal artery associated with high carotid bifurcation: a case report. Surg Radiol Anat. (2020) 42(9):1081–3. 10.1007/s00276-020-02452-4 32172358

[25] Shapiro R. Enlargment of the hypoglossal canal in the presence of a persistent hypoglossal artery. Radiology. (1979) 133(2):395–6. 10.1148/133.2.395 493525

[26] Udvarhelyi GB Lai M. Subarachnoid haemorrhage due to rupture of an aneurysm on a persistent left hypoglossal artery. Br J Radiol. (1963) 36:843–7. 10.1259/0007-1285-36-431-843 14080587

[27] Arnould G Tridon P Laxenaire M Picard L Weber M Gougaud G. The primitive hypoglossal artery. Anatomic and radio-clinical study. Apropos of 2 cases. Rev Neurol (Paris). (1968) 118(5):372–9. 5688566

[28] Nanto M Takado M Ohbuchi H Mandai A Osaka Y Nakahara Y Rare variant of persistent primitive hypoglossal artery, arising from the external carotid artery. Neurol Med Chir (Tokyo). (2012) 52(7):513–5. 10.2176/nmc.52.513 22850503

[29] Lee EJ Chang HW Cho CH Kim E Lee SK Kwon JH. Rare variant of persistent primitive hypoglossal artery in magnetic resonance angiography. Surg Radiol Anat. (2010) 32(8):801–4. 10.1007/s00276-010-0664-y 20390277

[30] Yamamoto R Mori N Nakae Y Tanaka F Johkura K. Anomalous anastomosis between the external carotid artery and vertebrobasilar artery via the hypoglossal canal: a case report and review of literature. Surg Radiol Anat. (2019) 41(7):849–52. 10.1007/s00276-019-02205-y 30729985

[31] Miller PA Oberg KC Sun A Achiriloaie A. A unique variant of a right persistent hypoglossal artery arising from the common carotid artery with complex cardiovascular anomalies in a female neonatal patient. J Radiol Case Rep. (2019) 13(9):28–35. 10.3941/jrcr.v13i9.3601 32184928 PMC7060012

[32] Minné C Du Toit J Jansen van Rensburg MN Mabiletsa MA Scheepers PA. Persistent primitive hypoglossal artery (a normal variant) as the sole supply to the brain. J Vasc Interv Radiol. (2012) 23(3):426–8. 10.1016/j.jvir.2011.11.031 22365302

[33] Sabouri S Ebrahimzadeh SA Rahimian N. Unusual variant of persistent primitive hypoglossal artery diagnosed by CT angiography: a case report and literature review. Clin Neuroradiol. (2014) 24(1):59–63. 10.1007/s00062-013-0201-6 23397245

[34] Fujihara F Takahara M Katsuta T Takemoto K Higashi T Inoue T. Posterior inferior cerebellar artery originating from the jugular branch of the ascending pharyngeal artery. NMC Case Rep J. (2019) 6(1):21–4. 10.2176/nmccrj.cr.2018-0171 30701151 PMC6350027

[35] Moffat DB Morris ED. Abnormal origin of the basilar artery from the cervical part of the internal carotid and its embryological significance. Anat Rec. (1956) 125(4):701–11. 10.1002/ar.1091250404 13362957

[36] Nakanishi N Sugino T Morikawa K Ohkawa N Fukusumi A. The posterior inferior cerebellar artery arising from the internal carotid artery directly: a variant of the persistent primitive hypoglossal artery. No to Shinkei. (2004) 56(3):253–7. 15112451

[37] Kim JT Heo SH Lee SH Choi SM Park MS Kim BC An uncommon anastomosis of the posterior inferior cerebellar artery and the external carotid artery with the patent vertebrobasilar system. Br J Radiol. (2009) 82(981):e171–4. 10.1259/bjr/73518980 19729545

[38] Uchino A Suzuki C. Variant of a persistent hypoglossal artery supplying only the posterior inferior cerebellar artery diagnosed by magnetic resonance angiography: a case report. Surg Radiol Anat. (2018) 40(7):807–10. 10.1007/s00276-018-1970-z 29330558

[39] Lasjaunias P Guibert-Tranier F Braun JP. The pharyngo-cerebellar artery or ascending pharyngeal artery origin of the posterior inferior cerebellar artery. J Neuroradiol. (1981) 8(4):317–25. 7328425

[40] Kimball D Ples H Miclaus GD Matusz P Loukas M. Persistent hypoglossal artery aneurysm located in the hypoglossal canal with associated subarachnoid hemorrhage. Surg Radiol Anat. (2015) 37(2):205–9. 10.1007/s00276-014-1285-7 24744136

[41] Shtadler DI Shtadler VD Staroverov MS Fukalov GA Karakulov OG Lebedev MA Cerebral persistent primitive arteries. Clinical case of combination with intracranial aneurysm and review of the literature. Zh Vopr Neirokhir Im N N Burdenko. (2024) 88(2):77–86. 10.17116/neiro20248802177 38549414

[42] De Caro R Parenti A Munari PF. The persistent primitive hypoglossal artery: a rare anatomic variation with frequent clinical implications. Ann Anat. (1995) 177(2):193–8. 10.1016/S0940-9602(11)80073-7 7741281

[43] Zhang L Chen X Jia L Dong L Wang J Liu P Case report: persistent primitive hypoglossal artery accompanied by a basilar bifurcation aneurysm treated by Y-stent-assisted coil embolization. Front Neurol. (2021) 12:621610. 10.3389/fneur.2021.621610 33746878 PMC7966712

[44] Yabuki R Baba EI Shirokane K Tsuchiya A Nomura M. Persistent primitive hypoglossal artery associated with multiple cerebral aneurysms. J Clin Med Res. (2019) 11(1):72–5. 10.14740/jocmr3649 30627281 PMC6306131

[45] Haryu S Shida N Tominaga T. Unusual case of persistent primitive hypoglossal artery with anterior choroidal artery aneurysm in Chiari type I malformation. Indian J Radiol Imaging. (2020) 30(3):383–5. 10.4103/ijri.IJRI_429_19 33273775 PMC7694719

[46] De Blasi R Medicamento N Chiumarullo L Salvati A Maghenzani M Dicuonzo F A case of aneurysm on a persistent hypoglossal artery treated by endovascular coiling. Interv Neuroradiol. (2009) 15(2):175–8. 10.1177/159101990901500206 20465895 PMC3299018

[47] Sawamura A Kamiyama H Kobayashi N Makino K Takizawa K Yasuda H Ruptured persistent primitive hypoglossal artery aneurysm: case report. No Shinkei Geka. (1999) 27(7):633–8. 10440037

[48] Baltsavias GM Chourmouzi D Tasianas N Drevelengas A Damianovski D Jovkovski S. Ruptured aneurysm of a persistent primitive hypoglossal artery treated by endovascular approach–case report and literature review. Surg Neurol. (2007) 68(3):338–43. 10.1016/j.surneu.2006.10.053 17719985

[49] Saito N Tanikawa R Tsuboi T Noda K Ota N Miyata S Posterior inferior cerebellar artery thrombosed aneurysm associated with persistent primitive hypoglossal artery successfully treated with condylar fossa approach. NMC Case Rep J. (2017) 4(3):93–6. 10.2176/nmccrj.cr.2016-0233 28840087 PMC5566692

[50] Kobayashi M Akaji K Tanizaki Y Mihara B Ohira T Kawase T. Posterior inferior cerebellar artery aneurysm associated with persistent primitive hypoglossal artery. Neurol Med Chir (Tokyo). (2008) 48(6):259–61. 10.2176/nmc.48.259 18574332

[51] Shigematsu H Yokota K Hirayama A Sorimachi T. Ruptured posterior inferior cerebellar artery aneurysm associated with persistent primitive hypoglossal artery: a case report. Radiol Case Rep. (2024) 19(1):146–9. 10.1016/j.radcr.2023.09.103 37941989 PMC10628796

[52] Yamamoto S Sunada I Matsuoka Y Hakuba A Nishimura S. Persistent primitive hypoglossal artery aneurysms–report of two cases. Neurol Med Chir (Tokyo). (1991) 31(4):199–202. 10.2176/nmc.31.199 1720206

[53] Hatayama T Yamane K Shima T Okada Y Nishida M. Persistent primitive hypoglossal artery associated with cerebral aneurysm and cervical internal carotid artery stenosis–case report. Neurol Med Chir (Tokyo). (1999) 39(5):372–5. 10.2176/nmc.39.372 10481441

[54] Huynh-Le P Matsushima T Muratani H Hikita T Hirokawa E. Persistent primitive hypoglossal artery associated with proximal posterior inferior cerebellar artery aneurysm. Surg Neurol. (2004) 62(6):546–51. 10.1016/j.surneu.2004.03.018 15576127

[55] Kanematsu M Satoh K Nakajima N Hamazaki F Nagahiro S. Ruptured aneurysm arising from a basilar artery fenestration and associated with a persistent primitive hypoglossal artery. Case report and review of the literature. J Neurosurg. (2004) 101(3):532–5. 10.3171/jns.2004.101.3.0532 15352614

[56] Genkai N Okamoto K Nomura T Abe H. Endovascular treatment of a ruptured aneurysm arising from the proximal end of a partial vertebrobasilar duplication with a contralateral prominent persistent primitive hypoglossal artery: illustrative case. J Neurosurg Case Lessons. (2021) 1(19):Case20108. 10.3171/CASE20108 35854835 PMC9245766

[57] Fujii Y Arai H Takeuchi S Sasaki O Kamada K Ogawa H An autopsy case of persistent primitive hypoglossal artery with multiple cerebral aneurysms. No Shinkei Geka. (1988) 16(4):421–6. 3386784

[58] Murumkar V Peer S Saini J Arvinda HR. Endovascular management of dissecting posterior cerebral artery aneurysm associated with persistent hypoglossal artery: a case report. J Vasc Bras. (2021) 20:e20200142. 10.1590/1677-5449.200142 34394204 PMC8336981

[59] Baldi S Zander T Rabellino M Maynar M. Stent-assisted coil embolization of a wide-neck aneurysm of a persistent primitive hypoglossal artery. Cardiovasc Intervent Radiol. (2009) 32(2):352–5. 10.1007/s00270-008-9415-4 18704567

[60] Hui FK Schuette AJ Cawley CM. Endovascular treatment of an aneurysm of a persistent primitive hypoglossal artery with complete resolution of brainstem compressive symptoms: case report. Neurosurgery. (2011) 68(3):E854–7. 10.1227/NEU.0b013e3182077d75 21311281

[61] Zeng S Yang D Yang H Xu LS Xu MH. A persistent primitive hypoglossal artery-posterior inferior cerebellar artery convergence aneurysm treated by stent-assisted coil embolization: a case report. Medicine (Baltimore). (2019) 98(39):e17151. 10.1097/MD.0000000000017151 31574820 PMC6775390

[62] He S Wei ML Xie F Richard SA. A fenestrated persistent primitive hypoglossal artery harboring a ruptured aneurysm: a case report. Medicine (Baltimore). (2021) 100(32):e26904. 10.1097/MD.0000000000026904 34397921 PMC8360458

[63] Kanazawa R Ishihara S Okawara M Ishihara H Kohyama S Yamane F. A successful treatment with carotid arterial stenting for symptomatic internal carotid artery severe stenosis with ipsilateral persistent primitive hypoglossal artery: case report and review of the literature. Minim Invasive Neurosurg. (2008) 51(5):298–302. 10.1055/s-0028-1082299 18855296

[64] Burgard M Psathas E Mordasini P Medlin F Menth M Egger B Symptomatic internal carotid artery stenosis in the presence of a persistent primary hypoglossal artery. Vascular. (2021) 29(4):543–9. 10.1177/1708538120966514 33175663

[65] Kadooka K Pamatmat R Ueda K Tsuboki S Mitsutake T Tanaka M. Symptomatic common carotid artery stenosis with a persistent primitive hypoglossal artery presenting with posterior circulation symptoms and technical challenges in stenting. Cureus. (2025) 17(4):e81562. 10.7759/cureus.81562 40182170 PMC11966179

[66] Eller JL Jahshan S Dumont TM Kan P Siddiqui AH. Tandem symptomatic internal carotid artery and persistent hypoglossal artery stenosis treated by endovascular stenting and flow reversal. J Neurointerv Surg. (2014) 6(4):e25. 10.1136/neurintsurg-2012-010578.rep 24719481

[67] Jin K Aihara N Tsukamoto T. A case of medial medullary infarction with persistent primitive hypoglossal artery. No to Shinkei. (2002) 54(4):341–5. 11993164

[68] Johnson MD Prestigiacomo CJ Ferioli S Flaherty ML. Persistent hypoglossal artery and concurrent carotid thrombus. Ann Neurol. (2020) 88(2):233–4. 10.1002/ana.25795 32447768

[69] Pinkerton JA Jr. Davidson KC Hibbard BZ. Primitive hypoglossal artery and carotid endarterectomy. Stroke. (1980) 11(6):658–60. 10.1161/01.STR.11.6.658 7210075

[70] Sunada I Yamamoto S Matsuoka Y Nishimura S. Endarterectomy for persistent primitive hypoglossal artery–case report. Neurol Med Chir (Tokyo). (1991) 31(2):104–8. 10.2176/nmc.31.104 1715037

[71] Ryu B Ishikawa T Hashimoto K Shimizu M Yagi S Shimizu T Internal carotid artery stenosis with persistent primitive hypoglossal artery treated with carotid artery stenting: a case report and literature review. Neuroradiol J. (2016) 29(2):115–21. 10.1177/1971400915626427 26825135 PMC4978312

[72] Iwaki K Arimura K Fukuda S Takagishi S Kurogi R Nakamura K Percutaneous transluminal angioplasty for persistent primitive hypoglossal artery stenosis: illustrative case. J Neurosurg Case Lessons. (2023) 6(17):CASE23427. 10.3171/CASE23427 37871338 PMC10599449

[73] Otomo Y Miyake S Akimoto T Nakai Y Oshio K Yamamoto T. Double distal filter protection with a single guiding catheter for internal carotid artery stenosis with a persistent primitive hypoglossal artery: a case report and literature review. J Neuroendovasc Ther. (2025) 19(1):cr-2025. 10.5797/jnet.cr.2025-0108 PMC12582895 41190288

[74] Shehab M Hoffmann RS Granbichler C Haddad M Bachar A. When carotid artery stenosis cause posterior Fossa infarct. An unusual case of persistent hypoglossal artery. Vasc Endovascular Surg. (2023) 57(8):919–22. 10.1177/15385744231183474 37294953

[75] Silva CF Hou SY Kühn AL Whitten RH Wakhloo AK. Double embolic protection during carotid artery stenting with persistent hypoglossal artery. J Neurointerv Surg. (2014) 6(3):e23. 10.1136/neurintsurg-2013-010709.rep 23661691

[76] Zhang L Song G Chen L Jiao L Chen Y Wang Y. Concomitant asymptomatic internal carotid artery and persistent primitive hypoglossal artery stenosis treated by endovascular stenting with proximal embolic protection. J Vasc Surg. (2016) 63(1):237–40. 10.1016/j.jvs.2014.04.066 24877853

[77] Yoshida S Kamatani K Takigawa K Tashiro N Hashiguchi Y Yasaka M Strategy of cerebral endovascular treatment for cervical internal carotid artery stenosis with a persistent primitive hypoglossal artery. Surg Neurol Int. (2023) 14:308. 10.25259/SNI_567_2023 37810314 PMC10559564

[78] Murai S Kusaka N Umakoshi M Itami H Otsuka S Nishiura T Stenting for internal carotid artery stenosis associated with persistent primitive hypoglossal artery using proximal flow blockade and distal protection system: a technical case report and literature review. J Stroke Cerebrovasc Dis. (2016) 25(6):e98–e102. 10.1016/j.jstrokecerebrovasdis.2016.03.026 27105567

[79] Hornung M Bertog SC Franke J Id D Grunwald I Sievert H. Evaluation of proximal protection devices during carotid artery stenting as the first choice for embolic protection. EuroIntervention. (2015) 10(11):1362–7. 10.4244/EIJY14M07_10 25042420

[80] Voronovich Z Grandhi R Zwagerman NT Jadhav AP Jovin TG. Manual aspiration thrombectomy for basilar infarction in the setting of a persistent primitive hypoglossal artery: case report and review of the literature. Surg Neurol Int. (2014) 5:182. 10.4103/2152-7806.147412 25593766 PMC4287913

[81] Uzawa A Aotsuka A Terano T. Posterior cerebral artery territory infarction associated with persistent primitive hypoglossal artery with internal carotid artery atherosclerosis. Intern Med. (2010) 49(5):515–6. 10.2169/internalmedicine.49.3075 20190498

[82] Kimura R Kato Y Kohyama S Suda S. Acute stroke associated with persistent primitive hypoglossal artery. Intern Med. (2025) 64(15):2422–4. 10.2169/internalmedicine.4806-24 39924237 PMC12393913

[83] Kawano H Inatomi Y Hirano T Yonehara T. Cerebral infarction in both carotid and vertebrobasilar territories associated with a persistent primitive hypoglossal artery with severe dilated cardiomyopathy. J Stroke Cerebrovasc Dis. (2014) 23(1):176–8. 10.1016/j.jstrokecerebrovasdis.2012.07.020 22959108

[84] Wu M Vedanayagam V White V. Simultaneous middle and bilateral posterior circulation stroke from persistent left hypoglossal artery. J Vasc Surg. (2025) 82(6):2251–2. 10.1016/j.jvs.2025.01.191 39889893

[85] See AP Baranoski JF Flores BC Ducruet A Albuquerque FC. Basilar stroke from a persistent hypoglossal artery. J Neurointerv Surg. (2017) 9(8):e30. 10.1136/neurintsurg-2016-012859.rep 28151414

[86] Han J Ji Y Ma G Kang Z. Recurrent cerebral infarction in anterior and posterior circulation territories associated with persistent primitive hypoglossal artery and carotid artery dissection: a case report. Int J Neurosci. (2018) 128(10):1003–5. 10.1080/00207454.2017.1408617 29166846

[87] Suzuki K Matsumaru Y Takeuchi M Morimoto M Kanazawa R Takayama Y Effect of mechanical thrombectomy without vs with intravenous thrombolysis on functional outcome among patients with acute ischemic stroke: the SKIP randomized clinical trial. JAMA. (2021) 325(3):244–53. 10.1001/jama.2020.23522 33464334 PMC7816103

[88] Fischer U Kaesmacher J SP P Bütikofer L Mordasini P Deppeler S SWIFT DIRECT: solitaire™ with the intention for thrombectomy plus intravenous t-PA versus DIRECT solitaire™ stent-retriever thrombectomy in acute anterior circulation stroke: methodology of a randomized, controlled, multicentre study. Int J Stroke. (2022) 17(6):698–705. 10.1177/17474930211048768 34569878

[89] Maïer B Finitsis S Bourcier R Papanagiotou P Richard S Marnat G First-line thrombectomy strategy for anterior large vessel occlusions: results of the prospective ETIS egistry. J Neurointerv Surg. (2022) 14(5):450–6. 10.1136/neurintsurg-2021-017505 33972458

[90] Patira R Kyper C Shah P Erkmen K. Bilateral persistent primitive hypoglossal arteries associated with unilateral symptomatic carotid thromboembolism. J Radiol Case Rep. (2017) 11(4):1–9. 10.3941/jrcr.v11i4.3010 28567180 PMC5439450

[91] Ishizuka T Endo H Yamaguchi S Hiratsuka Y Noro S Ishikawa K Endovascular treatment of acute atherothrombotic internal carotid artery occlusion associated with persistent primitive hypoglossal artery. Clin Neurol Neurosurg. (2024) 238:108179. 10.1016/j.clineuro.2024.108179 38387238

[92] Kwak R Emori T Yamamoto N Kadoya S. Moyamoya disease associated with persistent primitive trigeminal artery-report of two cases. No to Shinkei. (1983) 35(3):237–42. 6860501

[93] Katayama W Enomoto T Yanaka K Nose T. Moyamoya disease associated with persistent primitive hypoglossal artery: report of a case. Pediatr Neurosurg. (2001) 35(5):262–5. 10.1159/000050433 11741121

[94] Samra K Scoville WB Yaghmai M. Anastomosis of carotid and basilar arteries. Persistent primitive trigeminal artery and hypoglossal artery: report of two cases. J Neurosurg. (1969) 30(5):622–5. 10.3171/jns.1969.30.5.0622 5782389

[95] Wang M Gu J Lan P Wan S Zhou Y Zheng X A persistent primitive hypoglossal artery as the sole supply to the brain associated with a basilar bifurcation aneurysm. Front Neurol. (2017) 8:168. 10.3389/fneur.2017.00168 28491050 PMC5405127

[96] Kurose K Kishi H Sadatoh T. Moyamoya disease with persistent primitive hypoglossal artery. Case report. Neurol Med Chir (Tokyo). (1989) 29(6):528–32. 10.2176/nmc.29.528 2479858

[97] Li R Xiao X Yan Y Yu L Lv C Zhang Y GPRASP1 loss-of-function links to arteriovenous malformations by endothelial activating GPR4 signals. Brain. (2024) 147(4):1571–86. 10.1093/brain/awad335 37787182

[98] Yamanaka K Noguchi K Hayasaki K Matsuoka Y. Persistent primitive hypoglossal artery associated with arteriovenous malformation–case report. Neurol Med Chir (Tokyo). (1990) 30(12):949–55. 10.2176/nmc.30.949 1710324

[99] Yamauchi Y Aoyama T Sugitani Y Koama M Nukada T. Persistent hypoglossal artery associated with arteriovenous malformation–a study on cerebral circulation. Rinsho Shinkeigaku. (1974) 14(6):539–47. 4473299

[100] Gupta AK. Cerebral arteriovenous malformation embolized through persistent primitive hypoglossal artery: a case report. Interv Neuroradiol. (2005) 11(3):241–6. 10.1177/159101990501100307 20584481 PMC3404779

[101] Nishida C Ashikaga R Araki Y Nakamatsu K Ono Y Fujii K Persistent hypoglossal artery associated with arteriovenous malformation: a case report. Eur J Radiol. (2000) 33(1):59–62. 10.1016/S0720-048X(99)00058-3 10674792

[102] Pavlisa G Rados M Ozretic D Pavlisa G. Endovascular treatment of AVM-associated aneurysm of anterior inferior cerebellar artery through persistent primitive hypoglossal artery. Br J Neurosurg. (2012) 26(1):86–8. 10.3109/02688697.2011.584983 21707243

[103] Garza-Mercado R Cavazos E Urrutia G. Persistent hypoglossal artery in combination with multifocal arteriovenous malformations of the brain: case report. Neurosurgery. (1990) 26(5):871–6. 10.1227/00006123-199005000-00024 2352606

[104] Shibata Y Hyodo A Saito A Yoshii Y Nose T. Large arteriovenous malformation associated with persistent primitive hypoglossal artery–case report. Neurol Med Chir (Tokyo). (1991) 31(12):804–8. 10.2176/nmc.31.804 1726232

[105] Asayama K Hayashi K Kanai Y Tahara N Kato Y Abe S A case of coexistent persistent trigeminal and hypoglossal arteries manifested with neurovascular compression syndrome by hypertension. Brain Nerve. (2022) 74(6):811–6. 10.11477/mf.1416202125 35676216

[106] Meila D Wetter A Brassel F Nacimiento W. Intermittent hypoglossal nerve palsy caused by a calcified persistent hypoglossal artery: an uncommon neurovascular compression syndrome. J Neurol Sci. (2012) 323(1-2):248–9. 10.1016/j.jns.2012.08.018 23020989

[107] Elhammady MS Başkaya MK Sonmez OF Morcos JJ. Persistent primitive hypoglossal artery with retrograde flow from the vertebrobasilar system: a case report. Neurosurg Rev. (2007) 30(4):345–9. 10.1007/s10143-007-0092-6 17687575

[108] Garge S Moses V Keshava S Ahmed M Moorthy R. Persistent hypoglossal arteries with aneurysmal dilation of left hypoglossal artery: a rare case report and review of the literature. BJR Case Rep. (2016) 2(2):20150301. 10.1259/bjrcr.20150301 30363626 PMC6180872

